# Editorial—Medicinal Plants: Advances in Phytochemistry and Ethnobotany II

**DOI:** 10.3390/plants15020181

**Published:** 2026-01-07

**Authors:** Fabio Boylan, Dâmaris Silveira

**Affiliations:** 1 School of Pharmacy and Pharmaceutical Sciences, Faculty of Health Sciences, Panoz Institute, Trinity College, DO2 PN40 Dublin, Ireland; 2 Department of Pharmacy, Faculty of Health Sciences, University of Brasília, Brasília CEP 70910-900, DF, Brazil

The relationship between humankind and the environment has been pivotal to survival since *Homo sapiens*’ primordial ancestors. Plants remain the key to life as we know it. However, in the face of critical challenges such as global climate change, deforestation, urbanization, and the extinction of traditional languages or even entire communities [1,2], the collection and quality of Ethnobotany data is severely threatened, underscoring the urgent need for research in Ethnobotany and its related fields, such as Ethnopharmacology, Phytochemistry, Traditional Medicine, and Environmental Conservation. Ethnopharmacological research is crucial for understanding and preserving existing community knowledge, in addition to the changes in the new uses of plant resources. It also highlights the importance of environmental conservation in maintaining this delicate balance.

The global herbal medicine market is forecasted to be valued at USD 198.06 billion in 2025 and reach USD 326.46 billion by 2032, with a compound annual growth rate (CAGR) of 7.4% [3]. In addition to the rise in medicinal plant use, the increased search for cosmetics with herbal components and natural dietary supplements has driven this market’s continuous evolution [4].

A total of 18 manuscripts from across the world were submitted to this Special Issue of *Plants*, dedicated to Ethnobotany and Phytochemistry. Following a rigorous peer review process, seven papers were published, comprising Ethnobotany and Phytochemical aspects, primarily involving native species.

Aqueous extracts obtained from four Brazilian native species, *Cheiloclinium cognatum* (Miers) A.C. Sm. (Celastraceae), *Guazuma ulmifolia* Lam. (Sterculiaceae), *Hancornia speciosa* Gomes (Apocynaceae), and *Hymenaea stigonocarpa* Mart. ex Hayne (Fabaceae), were evaluated through an *in vitro* assay against acetaminophen toxicity. The extracts protected cells against acetaminophen’s deleterious effects, with protection ranging from 11% to 29%. Silymarin, used as a positive control, provided 38.2% protection against acetaminophen [5].

In another study, the radical scavenging, antimicrobial, and cytotoxic activities of an ethanol extract of *Lathyrus tuberosus* L. (Fabaceae), a species used as food, were investigated. Through the use of UPLC-MS/MS analysis, 93 compounds were identified, including flavonoids, lignans, iridoids, secoiridoids, and phenolic acid derivatives. The authors suggested that the high levels of polyphenols can, at least in part, explain the observed activities [6].

Three medicinal plants used in Traditional Medicine in Thailand—*Dracaena loureiri* Gadnep, *Ficus racemosa* Linn., and *Harrisonia perforata* (Blanco) Merr.—were evaluated against the A549 lung adenocarcinoma cell line. Ethanol extracts from *F. racemosa* and *H. perforata* were found to have a negligible effect. However, *D. loureiri* ethanol extract inhibited A549 cell growth, with an IC_50_ of 76.25 mg/mL, by inducing cell cycle arrest and apoptosis [7]. Another investigation showed that a xanthone isolated from a South American fruit tree—achachairu—presents hypotensive action in rats via endothelium-dependent vasorelaxant activity [8].

Plant derivatives play an essential role in pest and disease vector control. The effectiveness of essential oils from *Lonicera caprifolium* L. and *Pogostemon cablin* B. was demonstrated against the larvae of *Culex pipiens*, the vector of West Nile Virus (WNV), a causative agent of encephalitis. The authors also showed that nanoemulsions containing these essential oils were more active against the larvae than the pure plant derivatives [9].

In one compelling study, two species of *Monophadnus* (sawflies) were able to sequester phytoecdysteroids from their host plants. Furthermore, the authors investigated a furostenol saponin, revealing a dual functional role: it acts both as a defensive compound and a phagostimulant. Based on these findings, a combination of furostenol saponins and ecdysteroids is proposed for development as an effective repellent against specific insect pests [10].

Different plant propagation methods (greenhouse and micropropagation) were evaluated for *Zephyranthes irwiniana* (Ravenna) Nic. García, an endangered Brazilian species. Comparing traditional greenhouse techniques with *in vitro* micropropagation, the authors determined that micropropagation constitutes a superior propagation strategy. Crucially, they found that modifications to the culture medium not only improved biomass production but also positively influenced the profile and abundance of secondary metabolites [11].

Collectively, these studies underscore the significant potential of both classical phytochemistry processes and modern biotechnology in the discovery of valuable plant-derived compounds. In an era of accelerating climate change, which is already demonstrably altering global biodiversity cycles, the imperative to identify and study useful species, particularly endemic ones, has never been more urgent. The primary challenge and objective is to rapidly define and implement effective strategies for their conservation and sustainable exploration, thereby safeguarding these genetic and chemical resources for future generations.

In conclusion, it is imperative to emphasize that Ethnobotany and all biodiversity-driven research must adhere to rigorous best practices to ensure scientific accuracy, reproducibility, and ethical conduct throughout the investigative process. The implementation of established reporting guidelines such as the Consensus Statement on Ethnopharmacological Field Studies (ConSEFS) [12], the Consensus-based reporting guidelines for Phytochemical Characterization of Medicinal Plant extracts (ConPhyMP) [13], and other standardized techniques for ethnobotanical research [14] provides a critical framework. These tools are indispensable for fostering respectful and equitable collaboration between academics and traditional knowledge holders, ultimately leading to more robust, valid, and socially responsible scientific outcomes.
